# Design, fabrication, and evaluation of single- and multi-level 3D-printed non-covering cervical spinal fusion surgery templates

**DOI:** 10.3389/fbioe.2024.1416872

**Published:** 2024-07-12

**Authors:** A. H. Safahieh, H. Nazemi, N. Arjmand, P. Azimi, K. Khalaf

**Affiliations:** ^1^ Mechanical Engineering Department, Sharif University of Technology, Tehran, Iran; ^2^ Neuroscience Research Center, Shahid Beheshti University of Medical Sciences, Tehran, Iran; ^3^ Department of Biomedical Engineering and Health Engineering Innovation Center, Khalifa University of Science and Technology, Abu Dhabi, United Arab Emirates

**Keywords:** cervical spine, fusion surgery, template guides, pedicle screws, drilling, 3D-printing

## Abstract

**Background:**

Cervical spinal fusion surgeries require accurate placement of the pedicle screws. Any misplacement/misalignment of these screws may lead to injuries to the spinal cord, arteries and other organs. Template guides have emerged as accurate and cost-effective tools for the safe and rapid insertions of pedicle screws.

**Questions/Purposes:**

Novel patient-specific single- and multi-level non-covering templates for cervical pedicle screw insertions were designed, 3D-printed, and evaluated.

**Methods:**

CT scans of two patients were acquired to reconstruct their 3D spine model. Two sets of single-level (C3-C7) and multi-level (C4-C6) templates were designed and 3D-printed. Pedicle screws were inserted into the 3D**-**printed vertebrae by free-hand and guided techniques. For single-level templates, a total of 40 screws (2 patients × 5 vertebrae × 2 methods × 2 screws) and for multi-level templates 24 screws (2 patients × 3 vertebrae × 2 methods × 2 screws) were inserted by an experienced surgeon. Postoperative CT images were acquired to measure the errors of the entry point, 3D angle, as well as axial and sagittal plane angles of the inserted screws as compared to the initial pre-surgery designs. Accuracy of free-hand and guided screw insertions, as well as those of the single- and multi-level guides, were also compared using paired t-tests.

**Results:**

Despite the minimal removal of soft tissues, the 3D-printed templates had acceptable stability on the vertebrae during drillings and their utilization led to statistically significant reductions in all error variables. The mean error of entry point decreased from 3.02 mm (free-hand) to 0.29 mm (guided) using the single-level templates and from 5.7 mm to 0.76 mm using the multi-level templates. The percentage reduction in mean of other error variables for, respectively, single- and multi-level templates were as follows: axial plane angle: 72% and 87%, sagittal plane angle: 56% and 78%, and 3D angle: 67% and 83%. The error variables for the multi-level templates generally exceeded those of the single-level templates. The use of single- and multi-level templates also considerably reduced the duration of pedicle screw placements.

**Conclusion:**

The novel single- and multi-level non-covering templates are valuable tools for the accurate placement of cervical pedicle screws.

## 1 Introduction

Cervical spinal fusion, the procedure of fixing two or more vertebrae together, is a highly prevalent intricate surgical technique performed for the treatment of various spinal pathologies, including disc herniation (Lei et al., 2023), tumors (Goodwin et al., 2022), spinal instability (Pijpker et al., 2021), scoliosis (Cao et al., 2022), and trauma (Zuckerman et al., 2021). By the year 2040, the rate of cervical spine fusion surgeries is expected to increase by 14% reaching 209,000 operations per year in the United States alone (Neifert et al., 2020). This increasing trend can be attributed to multiple factors, such as aging (Halperin et al., 2024), trauma (Kapetanakis et al., 2024), sustained awkward postures (Alkosha et al., 2023), as well as overweight and obesity (Khan, 2023). A key factor behind the success of this surgery lies in the accurate placement of the screws inside the vertebral pedicles. Any misplacement/misalignment of these screws may lead to bone loss/weakening and substantial injuries to the spinal cord, arteries and other organs (Badiee et al., 2020). Designing an accurate and safe procedure for the placement of pedicle screws is, therefore, essential for ensuring adequate stability and pull-out force of the screws, as well as minimizing tissue injury during the operation (Bianco et al., 2017; Khatri et al., 2019).

Various approaches have thus far been suggested to optimize screw placements during the spinal fusion surgery, including fluoroscopy guided (Yanni et al., 2021), robotic-assisted (Davidar et al., 2024), 3D navigation (Chan et al., 2022; Bindels et al., 2024), and 3D-printed template guides. In order to evaluate these techniques, it is essential to consider several factors, such as the accuracy of pedicle screw insertion, reduced incidence of injury, and duration of the surgical procedure (Goldberg et al., 2022). The free-hand method has a low insertion accuracy, thus elevating the surgical risk particularly for the cervical pedicles with small cross-sectional areas (Modi et al., 2008; Parker et al., 2011). Accurate, fluoroscopy guided systems require multiple X-ray imaging during the surgery (Kochanski et al., 2019), thereby imposing considerable exposure risks to both patients and operating room staffs (Rampersaud et al., 2000). Similarly, robotic-assisted techniques produce acceptable accuracy, although their adaption is costly and limited to equipped healthcare facilities (Fatima et al., 2021). 3D navigation systems are also costly and require time-consuming registration procedures, which prolongs surgical duration and recovery (Liebmann et al., 2024). Therefore, 3D-printed template guides have emerged as accurate, cost-effective and available tools for the safe and rapid insertions of pedicle screws during spinal fusion procedures (Azimi et al., 2021).

Three main types of surgical template guides have been developed, including covering (Zhen-Shan et al., 2024), non-covering (Ma et al., 2023) and multi-level (Zhang et al., 2024) templates. An ideal surgical template should allow for the minimal removal of soft tissues, while maintaining maximal stability on the vertebrae throughout the drilling procedure. Covering templates are designed to conform to the vertebral surface, hence requiring the removal of all soft tissue from the bone (Garg et al., 2019; Pijpker et al., 2021). These templates have appropriate stability on their underlying vertebrae and provide great accuracy (Ribera-Navarro et al., 2021). Multi-level templates, on the other hand, offer some advantages in terms of reducing the complexity of use for fractured vertebrae, thereby decreasing surgical duration and ensuring adequate stability during the operation (Merc et al., 2013; Liu et al., 2017). However, these templates may cause screw misalignments due to changes between the position of the vertebrae observed in the pre-surgery CT images and their actual position during the surgery (Azimifar et al., 2017). Non-covering templates, through meticulous and optimal design, can achieve appropriate stability while minimizing contact with the vertebral bone, thus minimizing the need for soft tissue removal (Ashouri-Sanjani et al., 2021).

In continuation of our previous work on thoracic spine surgical templates (Ashouri-Sanjani et al., 2021), the objective of the present study is to design, fabricate, and evaluate patient- and vertebral-specific single- and multi-level non-covering templates for cervical pedicle screw insertions. The novel non-covering design aims to reduce duration of surgery, prioritize stability of the templates on the vertebrae, allow easy and accurate vertebral registration, and provide minimal invasiveness through reduced contact surface areas with the underlying bone. Toward this goal, CT scans of two patients were acquired and their 3D spine models were reconstructed. Two sets of single-level (C3-C7) and multi-level (C4-C6) templates were designed and 3D-printed. Pedicle screws were inserted into the 3D-printed vertebrae by two different techniques: free-hand and guided. For single-level templates, a total of 40 screws (2 patients × 5 vertebrae × 2 methods × 2 screws) and for multi-level templates 24 screws (2 patients × 3 vertebrae × 2 methods × 2 screws) were inserted by an experienced surgeon. Subsequently, CT images were acquired to measure the error of the entry point, 3D angle, as well as axial and sagittal plane angles of the inserted screws, as compared to the initial pre-surgery designs. Finally, accuracy of guided and free-hand screw insertions, as well as those of the single- and multi-level guides, were compared using paired t-tests. The success rate of the screw insertions was further assessed using the Gertzbein-Robbins classification (Gertzbein and Robbins, 1990). It is hypothesized that the employment of these novel surgical templates decreases the surgical duration and increases the accuracy of pedicle screw insertions.

## 2 Methods

**FIGURE 1:**
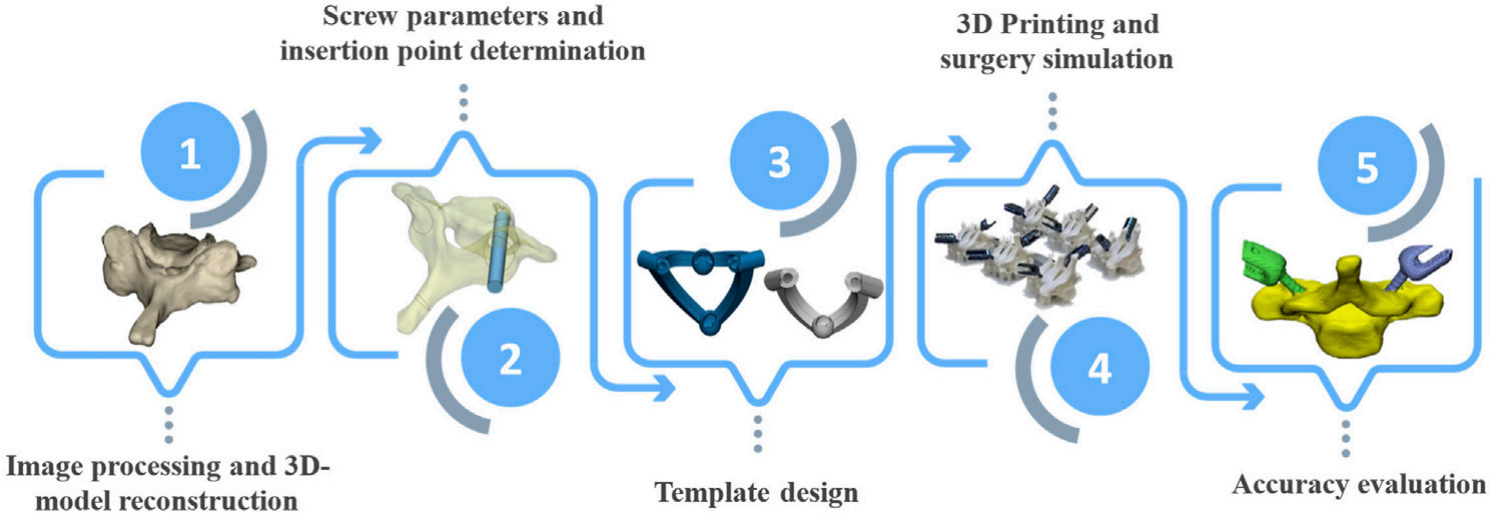
Flowchart of steps taken to design, fabricate, and test the cervical spine surgical templates.

The steps taken to design, fabricate, and test the cervical spine surgical templates are summarized in Figure 1 and detailed in the following sections.

### 2.1 Medical imaging

Two patients (a 37-year-old female and a 55-year-old male) underwent cervical spine CT scans using SIEMENS SOMATOM. In alignment with previous studies (Bundoc et al., 2017; Bundoc et al., 2023), the first two vertebrae of the cervical spine, C1 and C2, were excluded from this study due to their distinct geometry and surgical fusion approach as compared to other vertebrae. The CT images, with a slice thickness of 0.7 mm, were saved in a Digital Imaging and Communications in Medicine (DICOM) format, comprising a matrix of 511 × 511 pixels (pixel size for the first patient: 0.324 mm, second patient: 0.566 mm). A total of 455 CT images were acquired for these patients.

### 2.2 Image processing

**FIGURE 2:**
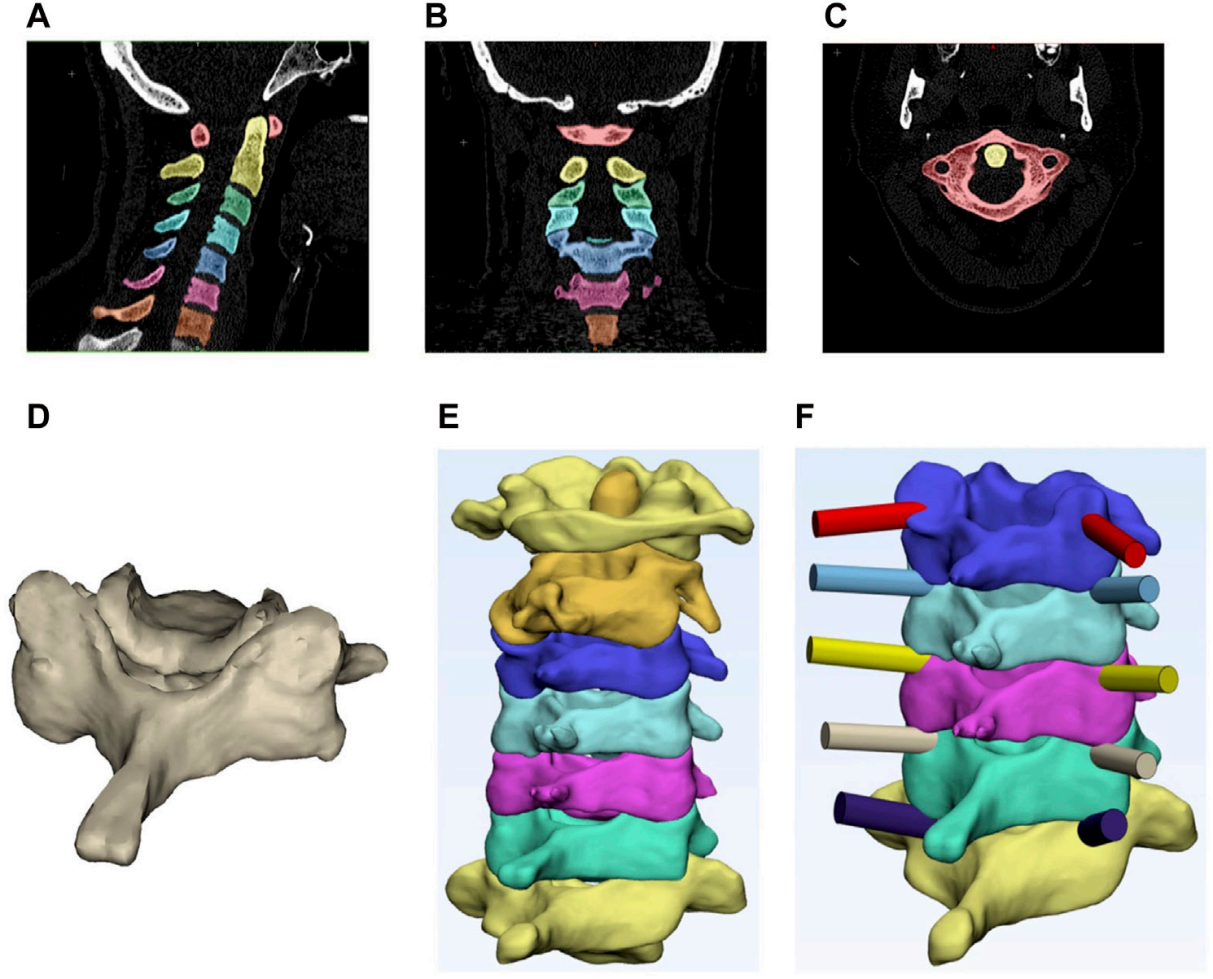
Top: segmented cervical vertebrae in the **(A)** sagittal plane, **(B)** frontal plane and **(C)** transverse plane. Bottom: **(D)** primary 3D reconstructed vertebra with no surface refinement, **(E)** smoothed 3D reconstructed model of the vertebrae, and **(F)** designed path for pedicle screw insertions.

An open-source software platform for medical images was employed to identify the boundaries of the vertebrae and perform 3D-model reconstruction. The Hounsfield unit threshold was set to 226-3071. To establish the 3D vertebral model, sections corresponding to the jaw, mastoid, and skull were removed from the CT images, retaining only the portions corresponding to the cervical spine (Figures 2A–C). Image noise reduction and gradient filters were applied to enhance the quality of the CT images. Subsequently, the vertebral boundaries were meticulously selected manually on a pixel-by-pixel basis. Following the creation of the 3D model, efforts were made to mitigate sharp corners and discontinuities. This involved the implementation of smoothing and surface refinement procedures on the vertebral model (Figures 2D, E).

### 2.3 Screw insertion parameters

**FIGURE 3:**
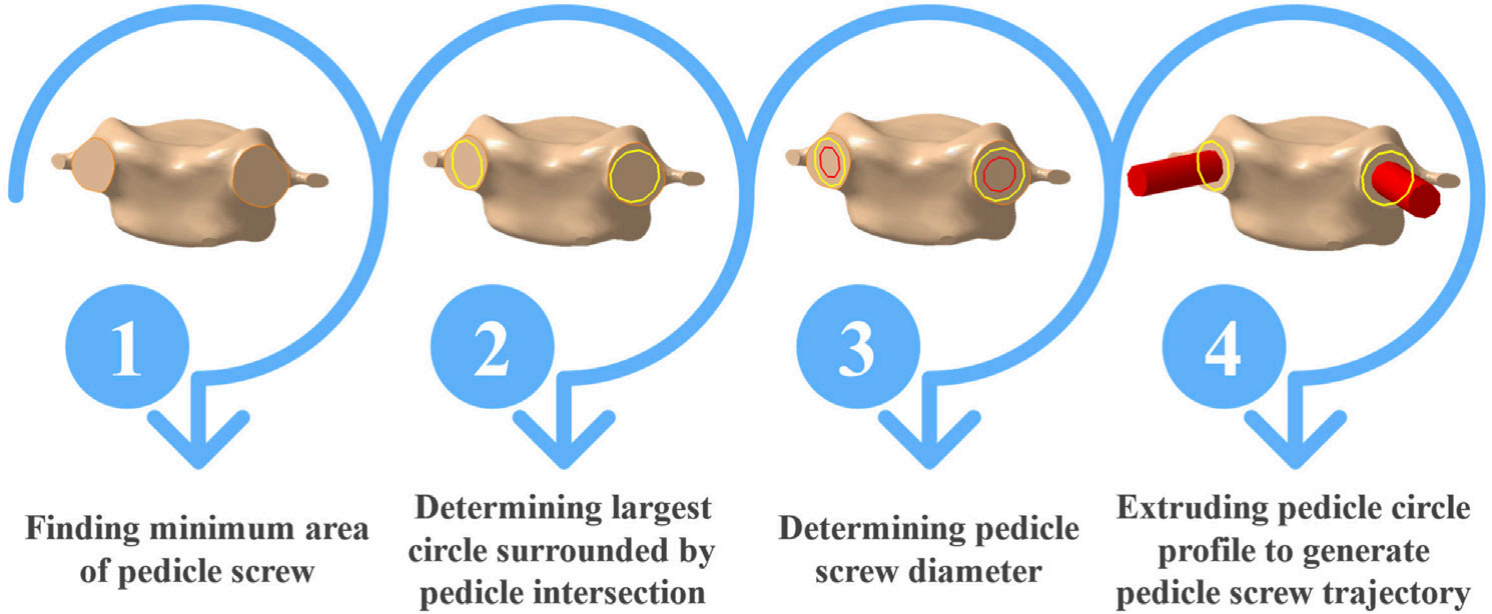
Flowchart of steps taken to find diameter and optimal trajectory of pedicle screws.

To identify the optimal trajectory of screws inside the vertebral pedicles, the minimum pedicle screw area needed to be determined. This involved the establishment of an imaginary plane intersecting with the pedicle (Figure 3, step 1). Subsequently, the maximal circle encapsulated within the minimum pedicle area was delineated (Figure 3, step 2), with the screw diameter set at 1–2 mm smaller than the diameter of this circumscribed circle (Figure 3, step 3). The trajectory and insertion point of the pedicle screw were next determined by extruding the pedicle screw profile, situated on the minimum cross-section plane of the pedicle in the normal direction (Figure 3, step 4). To optimize the bone-screw contact strength, the length of the screw was constrained not to exceed 90% of the vertebral body length (Dai et al., 2015). The identified optimal trajectory, represented as a cylinder (Figure 3, step 4), was then used as the gold standard path of the screw to evaluate postoperative insertion errors. Finally, the position of the cylinder relative to the vertebra was reassessed to ensure that the screw had no deviations, and the designed pathways were subjected to a throughout re-evaluation in collaboration with an experienced surgeon.

### 2.4 Design of single-level templates

The ease of registration on the vertebrae, high stability, and minimal contact surface represent the key characteristics of a suitable template. Facilitating easy vertebral template registration enables the surgeon to swiftly locate the template within the surgical site. Template instability during placement can lead to severe consequences, such as bleeding and injuries to vital organs. To ensure accurate template positioning, it is imperative to completely remove all soft tissues at the bone-template contact interface, ensuring the correct trajectory of the pedicle screw, while also providing sufficient stability. A minimal contact surface also reduces the time and energy required for detaching the soft tissues from the vertebrae and expediting the recovery time of patients. Templates, at their contact points, should feature a concave surface against the vertebrae, hence providing both stability and accuracy during pedicle screw insertions.

**FIGURE 4:**
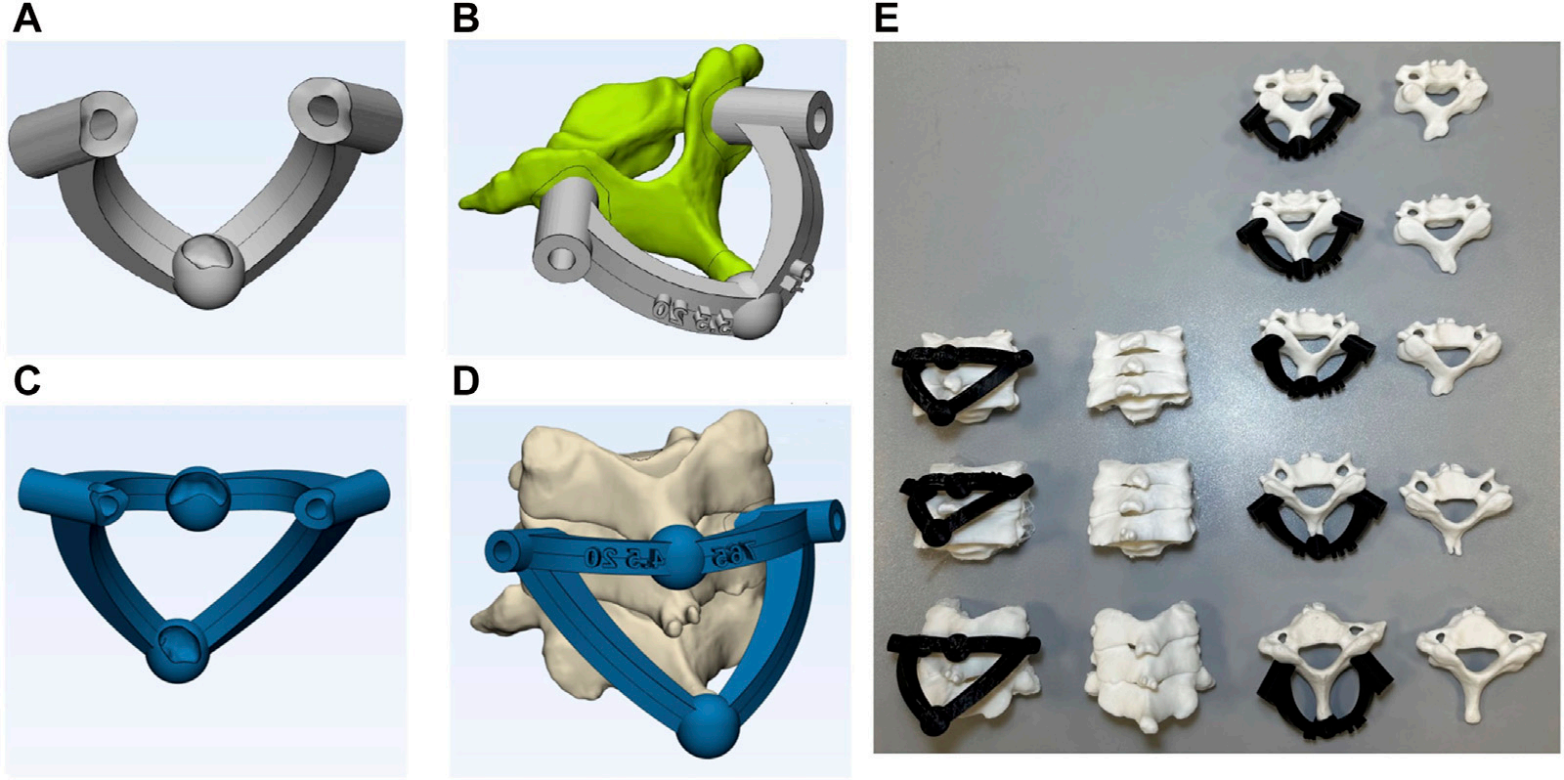
**(A)** single-level template, **(B)** single-level template located on vertebra, **(C)** multi-level template, **(D)** multi -level template located on vertebra, and **(E)** 3D-printed templates and vertebrae ready for the fusion surgery simulations.

In this study, the designed single-level template incorporated two contact surfaces at the screw insertion points into the vertebrae and one on the spinous process (Figures 4A, B). The spinous process was selected for its ease of identification by surgeons and thin, soft tissue covering. Following the refinement and surface smoothing of the reconstructed 3D model, the final geometry of the vertebrae was compared to the initial geometry to identify the areas which underwent the most significant changes. The results confirmed the accurate modeling of the spinous process, which remained largely unchanged during the refinement process. The spinous process was chosen as the third template contact point to ensure minimal alterations in the 3D-model during the surface refinement process. The third contact surface was designed as a sphere, featuring a concave surface in contact with the spinous process and serving as a convenient handle for the surgeon to push the template onto the vertebra. The radius of this sphere varied from 5 to 8 mm, depending on the vertebral size, to adequately cover the spinous process. The inner diameter of the template holes was designed to be equal to the screw diameter, while the outer diameter was 3 mm larger than the screw diameter. A total of 10 single-level templates were designed for the cervical vertebrae (C3 to C7) for both patients.

### 2.5 Design of multi-level templates

Multi-level templates are usually employed for the placement of screws in fractured vertebrae, where a direct template placement on the vertebra is difficult (Figures 4C, D). These templates utilize adjacent (upper and lower) vertebrae as the contact surfaces for screw placements. The effective use of these templates requires a fixed position of the adjacent vertebrae during both pre-surgery CT imaging and actual surgery (Azimifar et al., 2017). While the application of these templates on the thoracic and lumbar spines may pose some challenges due to stomach fullness and breathing, they are suitable for cervical surgeries due to their stable fixations. For multi-level templates, instead of using the spinous process of the corresponding vertebra as the contact surface, the spinous processes of the adjacent vertebrae are used as the third and fourth contact points, in addition to two insertion points on the pedicles. In this study, multi-level templates were designed for drilling the C4, C5 and C6 vertebrae for both patients thus resulting in a total of 6 templates.

### 2.6 3D-printing fabrication

Stereolithography (STL) files of the designed templates were extracted and underwent 3D-printing, utilizing the Fused Deposition Manufacturing (FDM) method (Figure 4E). The templates, crafted from black polylactic acid (PLA) with a thickness of 100 μm, were 3D-printed with a 100% filling rate using the Samin S3030V2 3D-printer. To assess accuracy of the pedicle screw placements between free-hand and template techniques, the vertebrae were also printed in two series.

### 2.7 Surgical simulation

**FIGURE 5:**
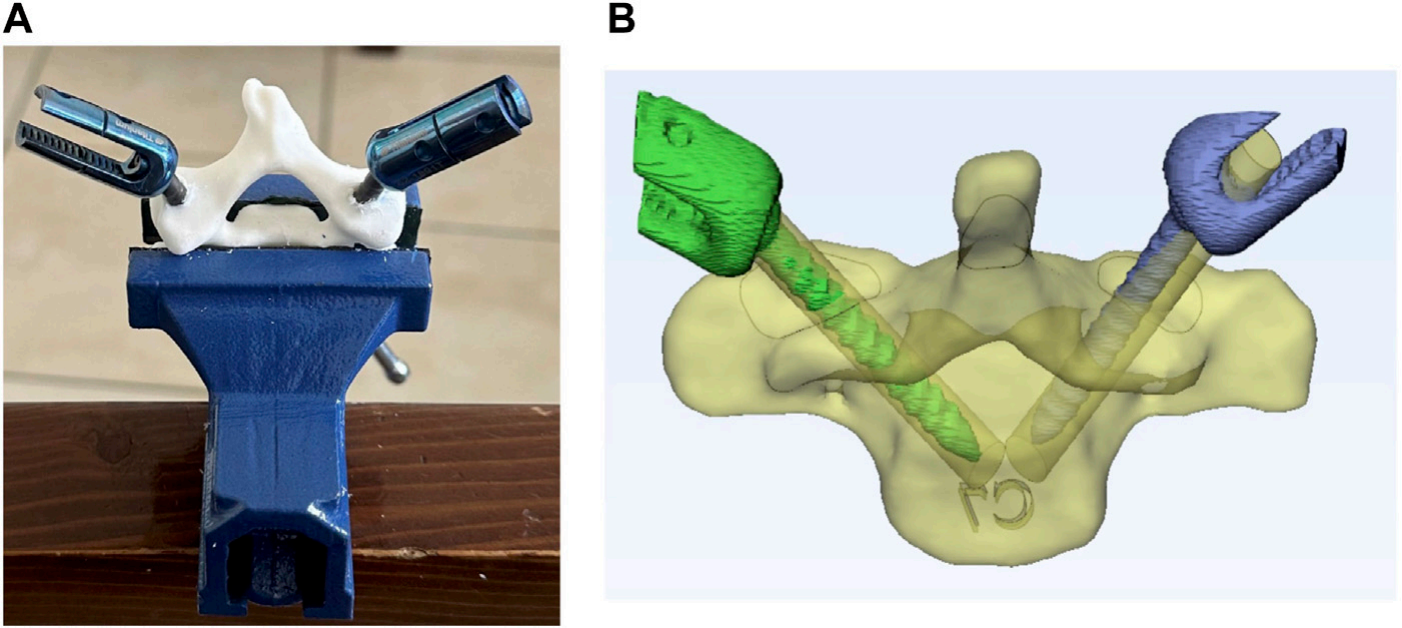
**(A)** Screws placed in vertebra and **(B)** reconstructed 3D-model of vertebra and screws for error evaluations.

For single-level and multi-level templates, respectively, one vertebra and three successive vertebrae were securely fixed within a holder. Pedicle screws (Osveh Asia Medical Instruments), with a 4.5 mm diameter and lengths of 25 and 30 mm, were expertly inserted by an experienced surgeon using two methods: free-hand and guided (Figure 5A). A total of 64 screws (32 screws for each patient) were inserted into the printed vertebrae (16 screws were placed by the free-hand technique and 16 by the surgical templates). For the guided placements, 10 screws were inserted using single-level templates, while 6 screws were placed using multi-level templates for each patient. To evaluate the performance of the templates in reducing surgical time, the total time of template registration on the vertebrae, as well as the time required for drilling in both single- and multi-level templates were recorded.

### 2.8 Accuracy evaluation

Post-surgery CT images (slice thickness of 0.1 mm) were acquired for all inserted screws. A 3D model was generated using an open-source medical image processing software (Figure 5B). The model was utilized to quantify the screw entry point error, 3D angle error, and angle error in both the sagittal and axial planes by comparison between the postoperative models and planned preoperative trajectories (Figure 2F). Statistical tests were employed to assess the improvement in the accuracy of screw placement using surgical templates. A comparison between the means of the two groups (single- and multi-level templates versus their own free-hand methods as well as between single- and multi-level templates themselves) was performed using paired t-tests for all the reported errors: entry point, 3D angle, and angle in both the sagittal and axial planes. The normality of error data was verified using the Shapiro-Wilk test at a significance level of 0.05. Gertzbein-Robbins classification (Gertzbein and Robbins, 1990) was also employed to examine the level of deviation in the placed screws.

## 3 Results

A total of 10 single- and 6 multi-level templates were meticulously developed and manufactured for screw insertions for two patients, resulting in the placement of 32 bilateral pedicle screws. Moreover, 32 screws were inserted via the conventional free-hand technique for benchmarking. Despite the relatively small bone-template contact surfaces, which mitigated the necessity for extensive soft tissue removal, the novel templates exhibited secure fixations on the vertebrae without any laxity. The concave-convex contact points, specifically the sphere encompassing the spinous process, enhanced the stability of the templates. In single-level templates, only one vertebra was fixed in the clamp, while in multi-level templates three successive vertebrae were placed together (Figure 4E). Insertion errors via the free-hand technique were reported separately for the single- and multi-level templates.

**FIGURE 6:**
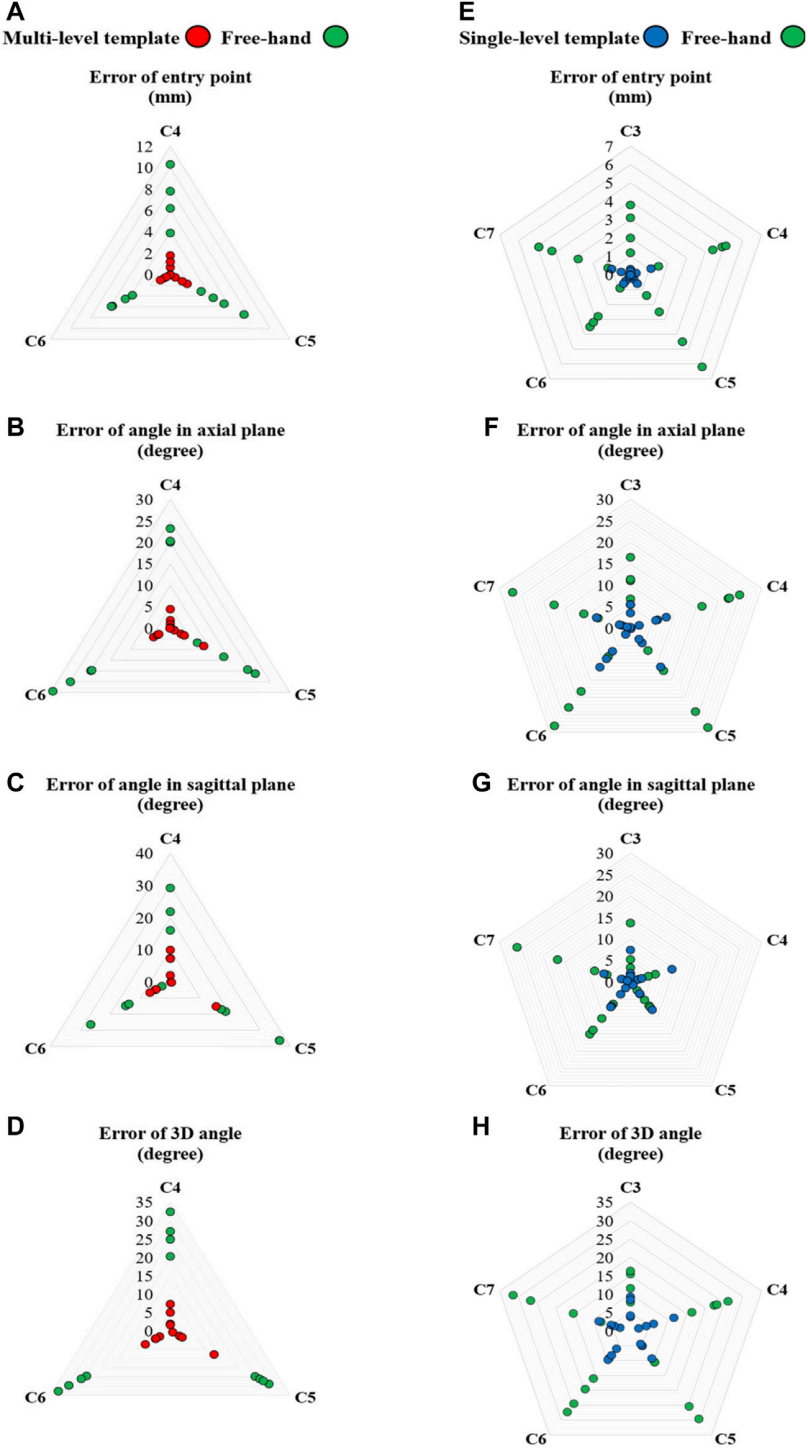
Radar plots of error variables for multi- (left) and single-level (right) templates *versus* the free-hand technique: Errors of **(A,E)** entry point, **(B,F)** angle in the axial plane, **(C,G)** angle in the sagittal plane and **(D,H)** 3D angle.

**FIGURE 7:**
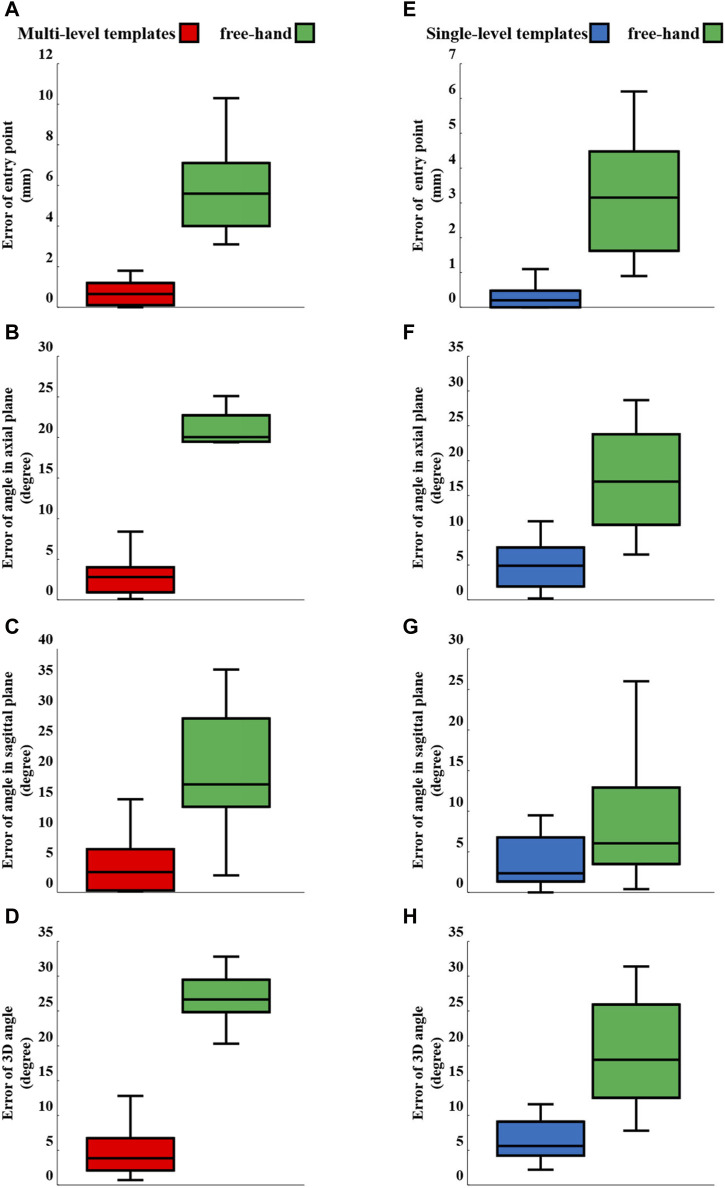
Box-plots of error variables for multi- (left) and single-level (right) templates *versus* the free-hand technique: Errors of **(A,E)** entry point, **(B,F)** angle in the axial plane, **(C,G)** angle in the sagittal plane and **(D,H)** 3D angle.

The Shapiro-Wilk tests confirmed the normality of the data. In all cases, the utilization of templates, when compared to the free-hand technique, yielded statistically significant reduction in the root mean square error (RMSE) and mean ± standard deviation of all the error variables, including entry point, axial plane angle, sagittal plane angle, and 3D angle for the pedicle screws (Tables 1, 2; Figure 6). While the mean values, standard deviation, and RMSE for entry point and angle in the sagittal plane error variables were smaller when using single-level templates as compared to multi-level templates (Tables 1, 2; Figure 6), the percentage of improvement relative to the free-hand technique was greater with multi-level templates in most error variable (Figure 7; Table 1).

**TABLE 1:** RMSE and mean ± standard deviation measurements of angles and entry point for single- and multi-level guided *versus* free-hand.

		Error of entry point (mm)	Error of angle in axial plane (degree)	Error of angle in sagittal plane (degree)	Error of 3D angle (degree)
Free-hand (single-level)	RMSE	3.54	18.71	10.10	20.50
	Mean ± standard deviation	3.02 ± 1.53	17.2 ± 7.57	8.01 ± 6.32	19.19 ± 7.40
Template (single-level)	RMSE	0.43	5.90	4.55	6.89
	Mean ± standard deviation	0.29 ± 0.33	4.87 ± 3.41	3.52 ± 2.94	6.38 ± 2.67
Free-hand(multi-level)	RMSE	6.02	20.60	22.49	27.28
	Mean ± standard deviation	5.70 ± 2.04	19.89 ± 5.61	20.08 ± 10.56	27.07 ± 3.51
Template(multi-level)	RMSE	0.97	3.54	6.35	5.53
	Mean ± standard deviation	0.76 ± 0.63	2.77 ± 2.31	4.46 ± 4.72	4.53 ± 3.31

**TABLE 2:** Paired *t*-test results to compare the errors between guided and free-hand techniques as well as between single-level and multi-level guides (* means significance of difference between the mean values).

Template	Error variable	*p*-value
Single-level *versus* free-hand	Entry point (mm)	<0.001*
	Angle in axial plane (degree)	<0.001*
	Angle in sagittal plane (degree)	0.008*
	3D angle (degree)	<0.001*
Multi-level *versus* free-hand	Entry point (mm)	<0.001*
	Angle in axial plane (degree)	<0.001*
	Angle in sagittal plane (degree)	<0.001*
	3D angle (degree)	<0.001*
Single-level *versus* multi-level	Entry point (mm)	0.048*
	Angle in axial plane (degree)	0.053
	Angle in sagittal plane (degree)	0.850
	3D angle (degree)	0.141

**FIGURE 8:**
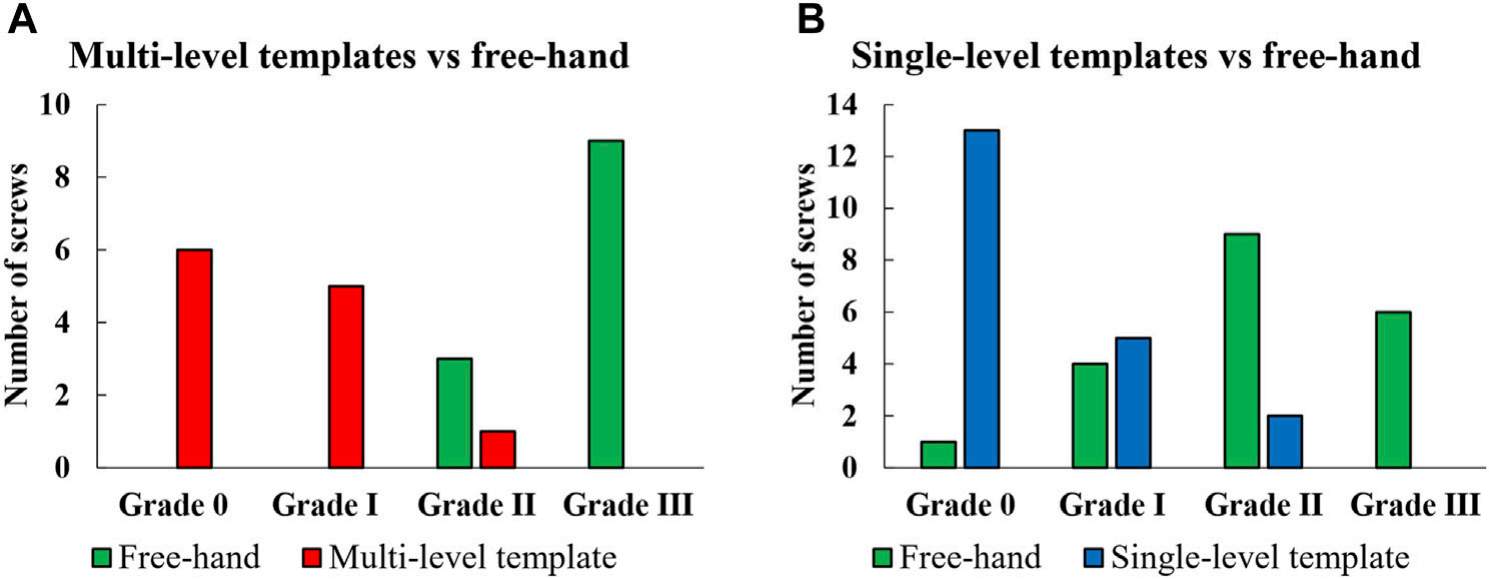
Comparison of multi- **(A)** and single-level **(B)** templates *versus* the free-hand technique according to Gertzbein-Robbins criteria.

Gertzbein-Robbins criterion indicated the absence of Grade III screws when using templates (Figure 8). Moreover, only 3 Grade II screws were observed when using template guides, i.e., 2 for single-level and 1 for multi-level templates (Figure 8). In contrast, the free-hand technique resulted in only 1 screw without any deviation. Finally, the required drilling time for each vertebra during the surgical simulation process indicated an average reduction of 69% and 73% in the drilling time for single- and multi-level templates, respectively (Table 3).

**TABLE 3:** Duration of pedicle screw insertion using single- and multi-level templates *versus* the free-hand techniques.

Single-level drilling				
Vertebra		Free-hand (Seconds)	Template (seconds)	Duration improvement (%)
C3		315	56	82
C4		165	63	62
C5		163	49	70
C6		154	59	62
C7		118	53	55
Average		183	56	69

Multi-level drilling			
Vertebra	Free-hand (Seconds)	Template (seconds)	Duration improvement (%)
C4	240	61	75
C5	202	58	71
C6	182	50	73
Average	208	56	73

## 4 Discussion

This study aimed to design, fabricate, and evaluate patient- and vertebral-specific single- and multi-level templates for cervical pedicle screw insertions. A total of 20 screws (10 screws per patient) and 12 screws (6 screws per patient) were, respectively, inserted using single- and multi-level templates. Additionally, 32 screws (16 screws per patient) were placed using the free-hand technique. The templates were designed based on 3D models of vertebrae reconstructed from CT images of the patients. Postoperative CT scans were also acquired to evaluate four quantitative error variables, alongside the qualitative evaluations, using the Gertzbein-Robbins criteria. Despite the minimal removal of soft tissues and contact surface, the findings revealed that the 3D-printed templates had acceptable stability and that their utilization led to a statistically significant reduction in all quantitative and qualitative errors. Moreover, while using the template guides required their placement on the vertebrae, the overall procedure time of the simulated surgery was shorter in the guided approach as compared to the free-hand approach.

The mean error of entry point using the single-level templates decreased from 3.02 mm (free-hand) to 0.29 mm; a reduction smaller than those reported by Wang et al. (0.8 mm) (Wang et al., 2018) and Sugawara et al. (0.7 mm) (Sugawara et al., 2017). Furthermore, the mean error of entry point using the multi-level templates decreased from 5.7 mm (free-hand) to 0.76 mm. The percentage reduction in mean of error variables for single- and multi-level templates, respectively, were as follows: entry point: 91% and 87%, axial plane angle: 72% and 87%, sagittal plane angle: 56% and 78%, and 3D angle: 67% and 83% (Table 1). Additionally, the utilization of single- and multi-level templates led to a reduction in the standard deviation across all error variables (Table 1; Figures 6, 7). The data points representing error variables associated with the 3D-printed templates were positioned closer, as compared to those of the free-hand approach, to the center of the plot (Figure 6). The utilization of the 3D-printed templates not only decreased error values but concentrated and made them more reproducible thereby mitigating the influence of surgeon experience on surgical outcomes.

According to the Gertzbein-Robbins classification, the percentage of acceptable inserted screws (i.e., Grade 0 and I), for free-hand technique was 25% (one vertebra fixed in the clamp), whereas it reached 90% when utilizing single-level templates (Figure 8). Additionally, the acceptability percentage for the free-hand technique was 0% (with three successive vertebrae fixed in the clamp), whereas it increased to 92% with the use of the multi-level templates. The measured error variables for the single-level and multi-level templates were generally insignificantly different except for the entry point in which the multi-level templates had significantly larger errors (Table 2). In cervical spine fusion surgery, the pedicle entry point is just below the facet joint and, if needed, the lower edge of the facet can be removed (Lau et al., 2017). However, this procedure was not included in the surgical simulation, serving as a rationale for limited reduction in the error of the entry point with the application of the multi-level templates compared to single-level templates. The mean of all error variables for the free-hand technique with one vertebra fixed in the clamp was lower compared to the case with three successive vertebrae fixed in the clamp (Table 1). Consequently, the percentage reduction in the mean error variable and RMSE for the multi-level templates was greater than those of the single-level templates, except for the error of entry point. For instance, although the mean error of the angle in the sagittal plane was lower for single-level templates as compared to multi-level templates (3.52 *versus* 4.46 degrees), the percentage improvement compared to the free-hand technique was higher for multi-level templates (56% *versus* 78%,) (Table 1). In cervical spinal surgeries, because of the small pedicle sizes and potential severe postoperative complications, the screws should be placed with the greatest possible accuracy. Therefore, based on our results, when surgery conditions allow, the use of single-level templates is recommended.

Notwithstanding the improvements in screw insertions by the templates, our study had some limitations. First, while only 64 screws were inserted in this study, the number of screws was sufficient to allow meaningful statistical analyses. Notably, several other studies with similar objectives used fewer or comparable number of screws: 57 screws (Wu et al., 2018), 48 screws (Sugawara et al., 2017), 8 screws (Kashyap et al., 2018), 48 screws (Deng et al., 2016), 74 screws (Guo et al., 2017), 48 screws (Kaneyama et al., 2014), 50 screws (Bundoc et al., 2017), 68 screws (Wang et al., 2019), 64 screws (Tian et al., 2019), and 68 screws (Pu et al., 2018). Second, the assessment of template stability and its locking mechanisms on the vertebrae was assessed qualitatively. This is a limitation shared with other investigations (Berry et al., 2005; Ma et al., 2012; Cecchinato et al., 2019; Ashouri-Sanjani et al., 2021; Ribera-Navarro et al., 2021), and should be addressed in future work. Third, the generalizability of the obtained results to actual surgeries might be carried out cautiously. Nevertheless, note that real surgeries impose conditions for the free-hand techniques that are as challenging as those encountered when using 3D-printed templates. It might therefore be argued that the utilization of these templates enhances the accuracy of pedicle screw placements and reduces the duration of placement in real surgical scenarios. Forth, the process involved in designing and fabricating the templates was time-consuming. In cases where rapid design of these templates is imperative, traditional methods are inadequate, necessitating an automated and systematic design approach for each patient and vertebra. Fifth, all pedicle screws were placed by a single surgeon and thus the potential impact of different surgeon experiences on the insertion accuracy was not assessed. Finally, the lack of diverse anatomies and pathological conditions; clinical trials used to assess postoperative complications associated with the use of template guides; quantitative assessment of stability of the templates, as well as neglecting soft tissue coverage over the 3D-printed vertebrae were further limitations of this study. Using template guides improved the accuracy of pedicle screw placement, thus decreasing the risk of screw loosening, as confirmed through pull-out tests in previous studies (Costa et al., 2013; Li et al., 2015). It is, therefore, expected that using template guides would also reduce the risk of revision surgeries, as well as potential complications, such as nerve and vein injuries. Nevertheless, applying template guides during fusion surgeries requires the removal of soft tissues from the underlying bone (vertebra), a procedure that may have adverse effects and postoperative complications despite the inherent thin soft tissue coverage of the cervical spine and our template design for the minimal contact area with the underlying vertebrae.

In conclusion, the surgical simulations conducted in this study demonstrated that both single- and multi-level templates reduced the duration of pedicle screw placement, RMSE, mean, and standard deviation of all error variables as compared to the free-hand technique. Considering the Gertzbein-Robbins classification, the number of grade 0 and I placements increased when the templates were implemented. These findings suggest that the novel templates hold promise for improving spinal fusion surgical outcomes. Our study had, however, some limitations such as the lack of diverse anatomies and pathological conditions; clinical trials to assess postoperative complications associated with the use of template guides; quantitative assessment of stability of the templates; as well as neglecting soft tissue coverage over the 3D-printed vertebrae. Future studies should incorporate the use of these templates, and for wider adoption, efforts should be directed towards automating the design process based on patient- and vertebra-specific basis.

## Data Availability

The original contributions presented in the study are included in the article/Supplementary Material, further inquiries can be directed to the corresponding author.
